# Human Chemokines as Antimicrobial Peptides with Direct Parasiticidal Effect on *Leishmania mexicana In Vitro*

**DOI:** 10.1371/journal.pone.0058129

**Published:** 2013-03-22

**Authors:** Sara K. Söbirk, Matthias Mörgelin, Arne Egesten, Paul Bates, Oonagh Shannon, Mattias Collin

**Affiliations:** 1 Division of Infection Medicine, Department of Clinical Sciences, Lund University, Lund, Sweden; 2 Clinical Microbiology, University and Regional Laboratories Region Skåne, Lund, Sweden; 3 Section for Respiratory Medicine and Allergology, Department of Clinical Sciences, Lund University, Lund, Sweden; 4 Division of Biomedical and Life Sciences, Faculty of Health and Medicine, Lancaster University, Lancaster, United Kingdom; University of Leuven, Rega Institute, Belgium

## Abstract

Chemokines and chemokine receptor-mediated effects are important mediators of the immunological response and cure in human leishmaniasis. However, in addition to their signalling properties for leukocytes, many chemokines have also been shown to act directly as antimicrobial peptides on bacteria and fungi. We screened ten human chemokines (CXCL2, CXCL6, CXCL8, CXCL9, CXCL10, CCL2, CCL3, CCL20, CCL27, CCL28) for antimicrobial effects on the promastigote form of the protozoan parasite *Leishmania mexicana*, and observed direct parasiticidal effects of several, CCL28 being the most potent. Damage to the plasma membrane integrity could be visualised by entrance of propidium iodide, as measured with flow cytometry, and by scanning electron microscopy, which showed morphological changes and aggregation of cells. The findings were in concordance with parasiticidal activity, measured by decreased mitochondrial activity in an MTT-assay. This is the first report of direct antimicrobial activity by chemokines on parasites. This component of immunity against *Leishmania* parasites identified here warrants further investigation that might lead to new insight in the mechanisms of human infection and/or new therapeutic approaches.

## Introduction

Cutaneous, mucocutaneous and visceral leishmaniasis in humans are caused by several species of the intracellular protozoan parasite *Leishmania.* The clinical picture and outcome of disease depends on the particular *Leishmania* species that causes infection, immunological status, and possibly also genetic factors of the host.

When the promastigote form of the parasite enters the haemorrhagic pool caused by the bite of an infected sand fly, it is rapidly taken up by dermal macrophages, neutrophils and other antigen presenting cells. Within macrophages, the promastigote transforms to the smaller amastigote form, which multiplies in the phagosome/parasitophorous vacuole of the macrophage. When the macrophage eventually ruptures, the amastigotes can be taken up by other macrophages and continue to divide. Throughout its life cycle, the *Leishmania* parasite has to withstand the immune systems of both vector and host long enough to secure further transmission through the next bite. It has therefore evolved different ways to modulate the immune response of the mammalian host outside and within the hostile environment of the phagosomes of macrophages.

In order for the host to clear infection, a cellular immune response is required, where a majority of the *Leishmania*-specific CD4+ T-cells differentiate into T helper (Th) 1-cells. The Th1-cells secrete IFN-γ, TNF-α, activate macrophages, and are important for parasite elimination. Many chemokines (CCL2, CCL3, CXCL8, CXCL9, CXCL10) are important as chemotactic agents and through their activation of different types of leukocytes in leishmaniasis [1]. There is also evidence that certain chemokines (CCL3, CCL4, CCL5, MCAF) are antiparasitic and increase phagocytosis and NO-mediated killing of different protozoa, but to date such effects have been attributed to indirect activity on the macrophages and not to a possible direct effect on the parasites [2], [3], [4], [5].

Infection with *Leishmania* parasites alters expression of chemokines and chemokine receptors in the host [6], [7]. *Leishmania major* has been shown to up-regulate genes for IL-8, CXCL2, CXCL3,CCL20 and down-regulate CXCL9, CXCL10 and CCL2 in human macrophages in vitro [7], possibly part of an immune evasion response. Conversely, in skin lesions taken from patients with localised (typically self-healing) cutaneous leishmaniasis, CXCL9, CXCL10 and CCL2 are highly expressed, and CCL3 is only present in low levels, this response perhaps being part of an effective immune response. Supporting this idea, in the more chronic and severe diffuse forms of cutaneous leishmaniasis, the former chemokines are expressed in low levels, and there are high amounts of CCL3 [8], [9]. These and other data imply that chemokines are important players in immunity against *Leishmania* parasites.

Antimicrobial peptides (AMPs) and chemokines have many properties in common; they are small, cationic proteins, and both groups are believed to be important components of the innate immunity in a wide range of species. They are usually expressed in low levels, but up-regulated in response to infectious or inflammatory stimuli [10]. The AMPs in the groups of α- and β-defensins are chemotactic for leukocytes. β-defensins share their receptor, CCR6, with the chemokine CCL20, which has also been shown to have direct antimicrobial activity [11], [12]. A number of chemokines have, in addition to their different effects on human cells, been shown to have antimicrobial activities on bacteria and fungi (Table 1), and sometimes the term kinocidins is used for these molecules [13], [14], [15]. Further, several AMPs from various species are reported to have effects on different *Leishmania* species [16]. The virulence factor Gp63, a multifunctional metalloprotease prominent on the surface of promastigotes, also protects against antimicrobial peptide-induced killing of *Leishmania major* [17].

**Table 1:** Chemokines used in this study and previous reports of their antimicrobial activity.

Chemokine new/old name	Main leukocyte targets	Antimicrobial activity	Calculated pI
CXCL2/GRO-β	Neutr, Mo, Mast	*E. coli, S. aureus * [*12*]	10.27
CXCL6/GCP-2	Neutr, Mo, Mast	*S. pyogenes, S. aureus, E. coli, P. aeruginosa,* *S. dysgalactiae* [26]	9.06
CXCL8/IL-8	Neutr, Mo, Mast	*S. aureus, S. typhimurium, C. albicans * [*13*]	8.97
CXCL9/MIG	Th1, NK, pDC, Mast	*E. coli, S. aureus, S. pyogenes, N. gonorrhoeae* [12]	10.83
CXCL10/IP-10	Th1, NK, pDC, Mast	*E. coli, S. aureus* [12]	10.52
CCL2/MCP-1	Mo, Bas, MemT	No activity on *E. coli*, *S. aureus* [12]	9.58
CCL3/MIP-1α	mDC, Mo, MemT, Th1, Treg, NK, pDC	No activity on *E. coli*, *S. aureus* [12]	4.60
CCL20/MIP-3α	BC, mDC, MemT	*E. coli, S. aureus, C. albicans * [*12*]	10.08
CCL27/CTACK	MemT	*C. albicans*. No activity on *E. coli, S. aureus* [12], [27]	9.11
CCL28/MEC	MemT	*S.pyogenes, S.aureus, E.coli, C.albicans * [*27*]	10.23

Chemokines are listed with their systematic/common names, main leukocyte targets [23] and antimicrobial activity previously reported. Bas, basophils;BC, B-cells; Mast, mast cells; mDC, myeloid dendritic cells; MemT, memory T-cells; Mo, macrophages; NK, natural killer cells; Neut, Neutrophils; pDC, plasmacytoid dendritic cells; Th1, T-helper 1 cells. Calculated pI may be important, as the cationic properties of the peptides, at least in part, may explain the interaction with the plasma membrane and the antimicrobial activity. Theoretical pI for CCL28 was computed using the EXPASy primary structure analysis tool, others taken from Yang et al. 2003 and Svensson et al. 2010 [12], [28].

To our knowledge, there are no previous reports of chemokines having direct antimicrobial effects on parasites, independent of the activation of leukocytes. As several chemokines have previously been shown to exhibit antimicrobial effects on both bacteria and the eukaryote *Candida albicans*, our aim was to screen the most probable candidates for possible effects on a protozoan parasite, *Leishmania mexicana*. For this study, we chose chemokines previously tested for antimicrobial effect, some of which had also been shown to be up or down regulated in leishmaniasis, and here we present the evidence for antiparasitic activity of several of them.

## Results

### Several Chemokines Decrease the Mitochondrial Activity of L. mexicana

**Figure 1:**
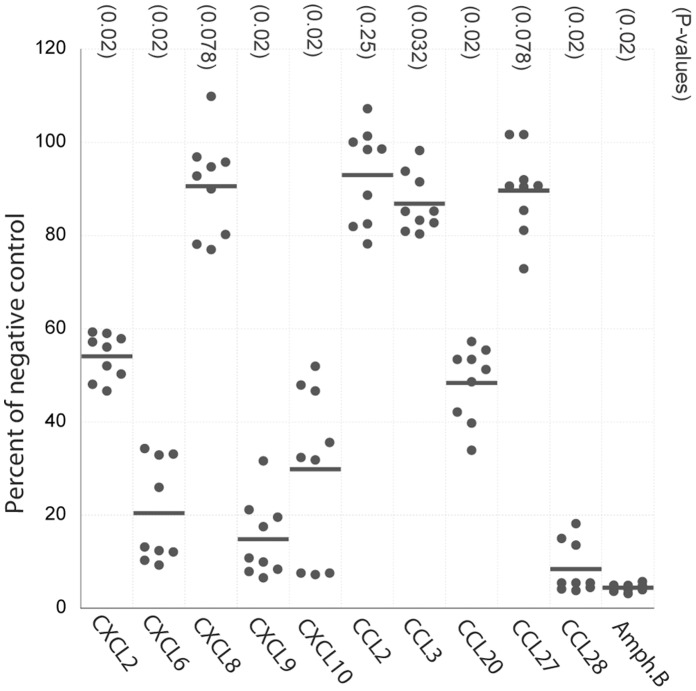
Cytotoxic activity of chemokines against *Leishmania* promastigotes. Reduction of mitochondrial activity in the MTT assay, as a measure of cytotoxic activity of the chemokines on the promastigotes after 4 hrs of incubation with 10 µM of the chemokines or 5 µM of Amphotericin B. The MTT assay was performed in three independent experiments on different days, and each incubation performed in triplicate. Horizontal lines represent mean values of individual measurements and are expressed as percentages of negative controls. P-values (for significant difference to negative control) were calculated using the Wilcoxon Rank-Sum test, adjusted with the Hochberg procedure for multiple comparisons.

**Figure 2:**
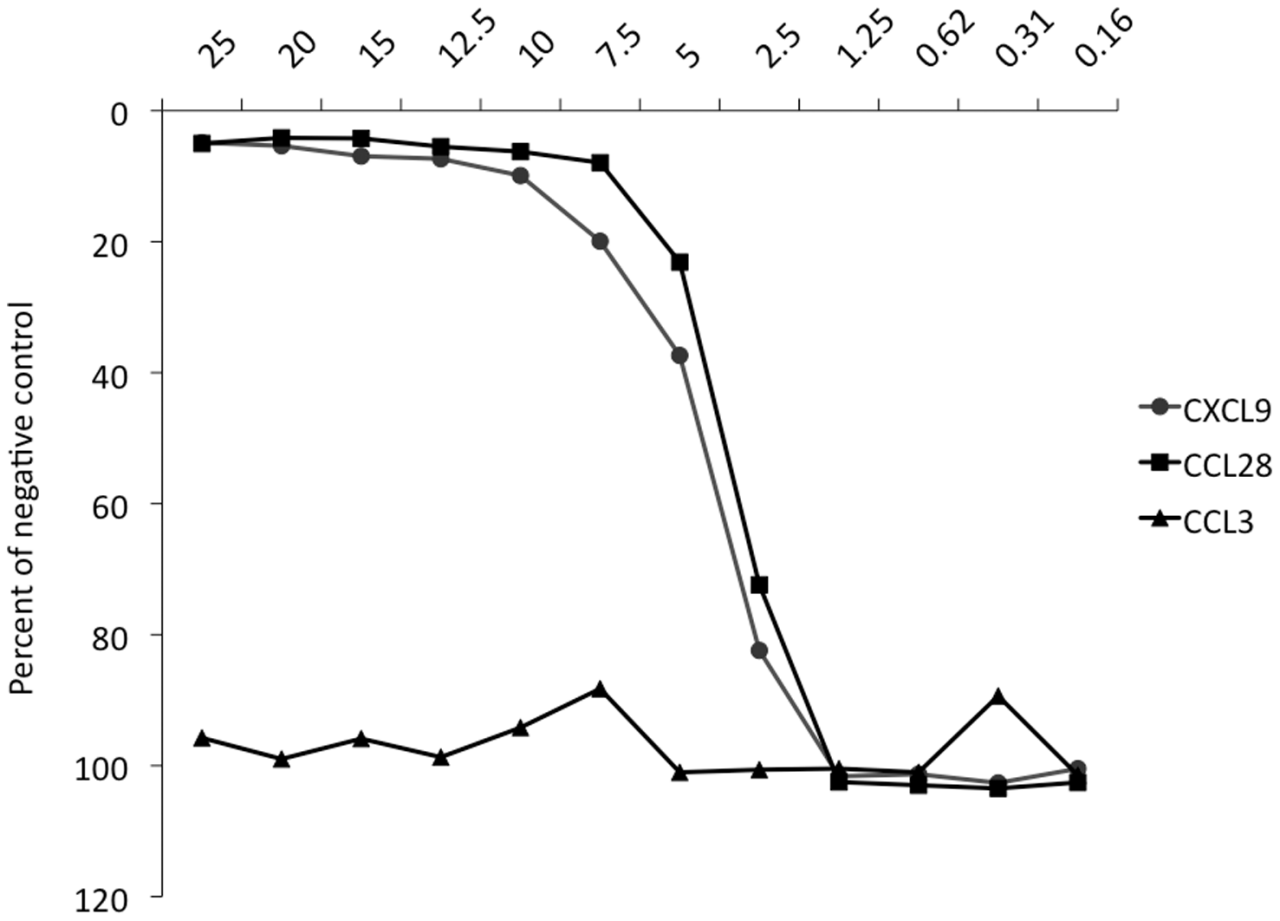
Dose-response of CXCL9 and CCL28 on promastigotes. Reduction of mitochondrial activity in the MTT-assay, as a measure of cytotoxic activity of CXCL9 and CCL28 on the promastigotes after 4 hrs of incubation with the chemokines in concentrations from 25 to 0.16 µM, with CCL3 for comparison, all assayed against negative controls. A dose-dependent response can be seen between 15 and 1,25 µM for both CXCL9 and CCL28.

Cytotoxicity was measured in an MTT-based assay, in which viable actively respiring cells with functional mitochondria convert 3-(4,5-Dimethylthiazol-2-yl)-2,5-Diphenyltetrazolium Bromide (MTT) to purple formazan, measured in a spectrophotometer. This method, which has been previously validated for *Leishmania* parasites in both HBSS and sorbitol buffer systems [18], [19], and in our settings (Fig. S1), gives a measure of viable cells, but not the mechanism of the cytotoxic effect. In three identical independent experiments, cytotoxic activity was observed for several of the chemokines that have previously shown antibacterial and antifungal properties (Fig. 1). CXCL6, CXCL9 and CCL28 were the most effective causing over 80% loss of viability, but substantial decrease of mitochondrial activity (over 50%) was also observed for CXCL2, CXCL10, and CCL20 (Table 2). Dose-response assays were carried out for CXCL9 and CCL28, which showed a dose-dependent activity for both (Fig. 2). CCL3 exerted a marginal effect, whereas CXCL8, CCL2 and CCL27 did not cause a statistically significant decrease in viability. These findings were in concordance with pilot studies in which cells were manually counted in a Neubauer chamber on days 0–3 after incubation with the chemokines, corresponding well to survival of cells on day 3 (Table S1). The most effective chemokines had an activity comparable to that of Amphotericin B, a currently deployed first-line antileishmanial drug that acts by forming pores in the surface membrane. Additional assays, also performed in sorbitol buffer with serum, or in HBSS (Hanks Balanced Salt Solution) with or without serum did not show the same activity as when performed in sorbitol buffer alone (Fig. S2, S3), but point toward a possible effect in HBSS with serum.

**Table 2:** Overview of observed activity on the promastigotes by the chemokines.

Chemokine	Cytotoxic activity	Membrane damage	Aggregation of cells	Morphologic change in SEM
CXCL2	++	++	+	+
CXCL6	+++	+++	+	+
CXCL8	−	−	−	N.D.
CXCL9	+++	++	−	+
CXCL10	++	++	−	−
CCL2	−	−	−	+
CCL3	−	−	−	N.D.
CCL20	++	+	++	+
CCL27	−	−	+	+
CCL28	+++	++	+++	+
Amph.B	+++	++	−	+

Cytotoxic activity in the MTT-assay (quantified – to +++), membrane damage measured by entry of PI in flow cytometry (quantified – to +++), aggregation of cells observed by the eye and in light microscopy (semiquantified – to +++), and morphologic changes of the cells visible in SEM (- or +) are summarized. N.D.  = not done.

### Chemokines Make Leishmanial Membranes Permeable to Vital Dyes

Membrane integrity can be assessed by the entry of vital dyes, and entry of propidium iodide (PI) has previously been used to study membrane damage in *Leishmania*.

**Figure 3:**
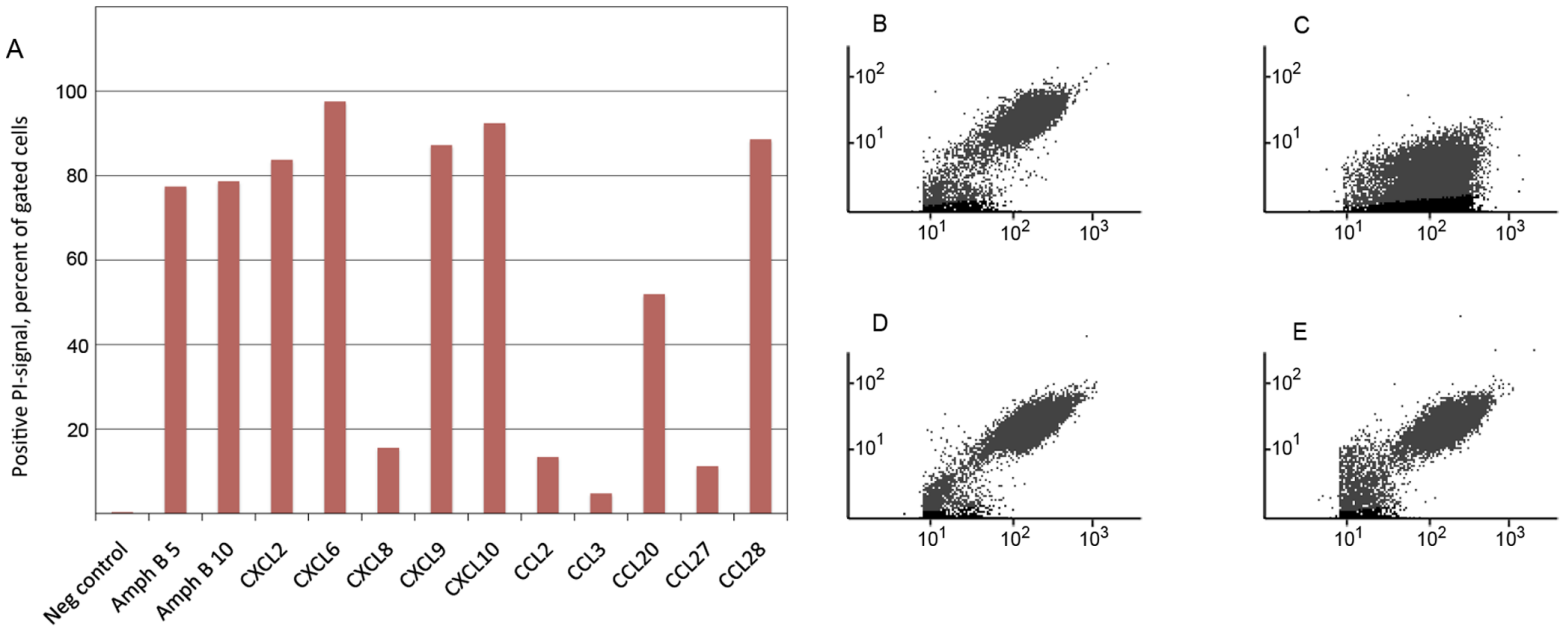
Damage to the promastigote surface membrane visualised by flow cytometry. PI-signal in flow cytometry, after incubation with chemokines, positive cells as a percentage of gated cells (A). Plots showing side scatter (*y*-axis) and forward scatter (*x*-axis) of untreated promastigote cells (B), and cells treated with Amphotericin B (C), CXCL9 (D) and CCL28 (E). Scatter plots (B-E) represent the whole population of (ungated) cells.

Flow cytometry was used to detect entrance of PI and after 4 hours of incubation, the plasma membranes of *L. mexicana* promastigotes were sufficiently permeabilized by several of the chemokines to allow entrance of PI (Fig. 3), a molecule of relative molecular mass 668 Da. Flow cytometry dot plots of cell morphology (forward scatter versus side scatter) revealed that the chemokines did generate some, but not gross morphology changes in the cells (Fig. 3B–E). With few exceptions, the chemokines yielding the highest percentage of PI-positive cells also showed the largest decrease in mitochondrial function in the MTT-assay, indicating membrane damage as a likely cause of the cytotoxic activity of the chemokines (Table 2).

### Chemokines Cause Aggregation of Promastigote Cells

When promastigotes were incubated with 10 µM of the chemokines in 1.5 mL tubes, an immediate aggregation of cells was visible for many of the chemokines. The aggregation of cells could be observed by the eye, and in light microscopy, where apparently intact cells were clumped together with morphologically altered cells and cell debris. Not all chemokines caused aggregation, and the degree of aggregation varied among those that did (CXCL2, CXCL6, CCL20, CCL27, CCL28; Table 2).

### Chemokines Cause Morphological Changes by Scanning Electron Microscopy (SEM)

**Figure 4:**
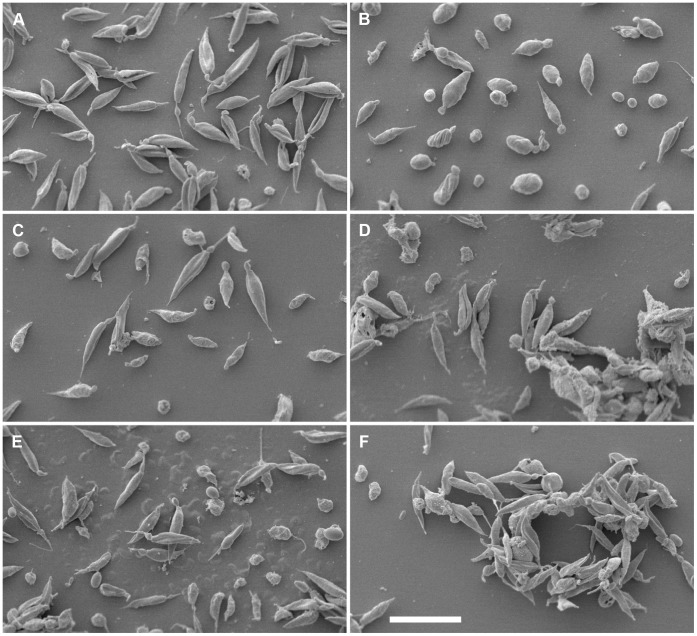
Damage to *Leishmania* promastigotes visualised by SEM. In scanning electron microscopy, untreated parasites retain their elongated and fusiform shape (A), whereas treatment with amphotericin B, known to act on plasma membranes, causes the promastigotes to round up and become smaller (B). This effect was also visible to varying degrees on promastigotes treated with many of the chemokines, here shown for CXCL9 (C), CCL20 (D), CCL27 (E) and CCL28 (F). Several of the chemokines caused aggregation when incubated with the parasites, here shown for CCL20 (D) and CCL28 (F), as was visible immediately after adding the chemokines when the incubation was made in 1.5 mL tubes (data not shown).

Previous studies have shown morphological changes of *Leishmania* promastigotes when treated with AMPs [17]. Electron microscopy has been used for *Leishmania* and other protozoan parasites to visualize cell surface alterations, cell shrinkage, and changes of cytoskeleton as an effect of antiparasitic chemotherapy [20]. In order to see if the chemokines used in our screen were inducing morphological changes, and in order to visualise the aggregations observed, SEM was used to examine the chemokine-treated promastigotes. In SEM (Fig. 4), promastigotes showed different degrees of morphological changes. Many cells exhibited shrinking/rounding up responses in a similar way as is seen after treatment with Amphotericin B, itself known to act on plasma membranes. This type of morphological effect on *Leishmania* promastigotes has previously been shown for antimicrobial peptides [17].

The aggregations which were visible macroscopically within seconds of incubating the parasite with some of the peptides were also visible in SEM for most of the chemokine-treated parasites (CXCL2, CXCL6, CCL20, CCL28). Aggregations of apparently intact and broken promastigotes were visible, sometimes with a matrix or fibrillar network of possible cytoplasmic or nuclear contents.

### Parasiticidal Chemokine CCL28 is not Cytotoxic to Human Cells

**Figure 5:**
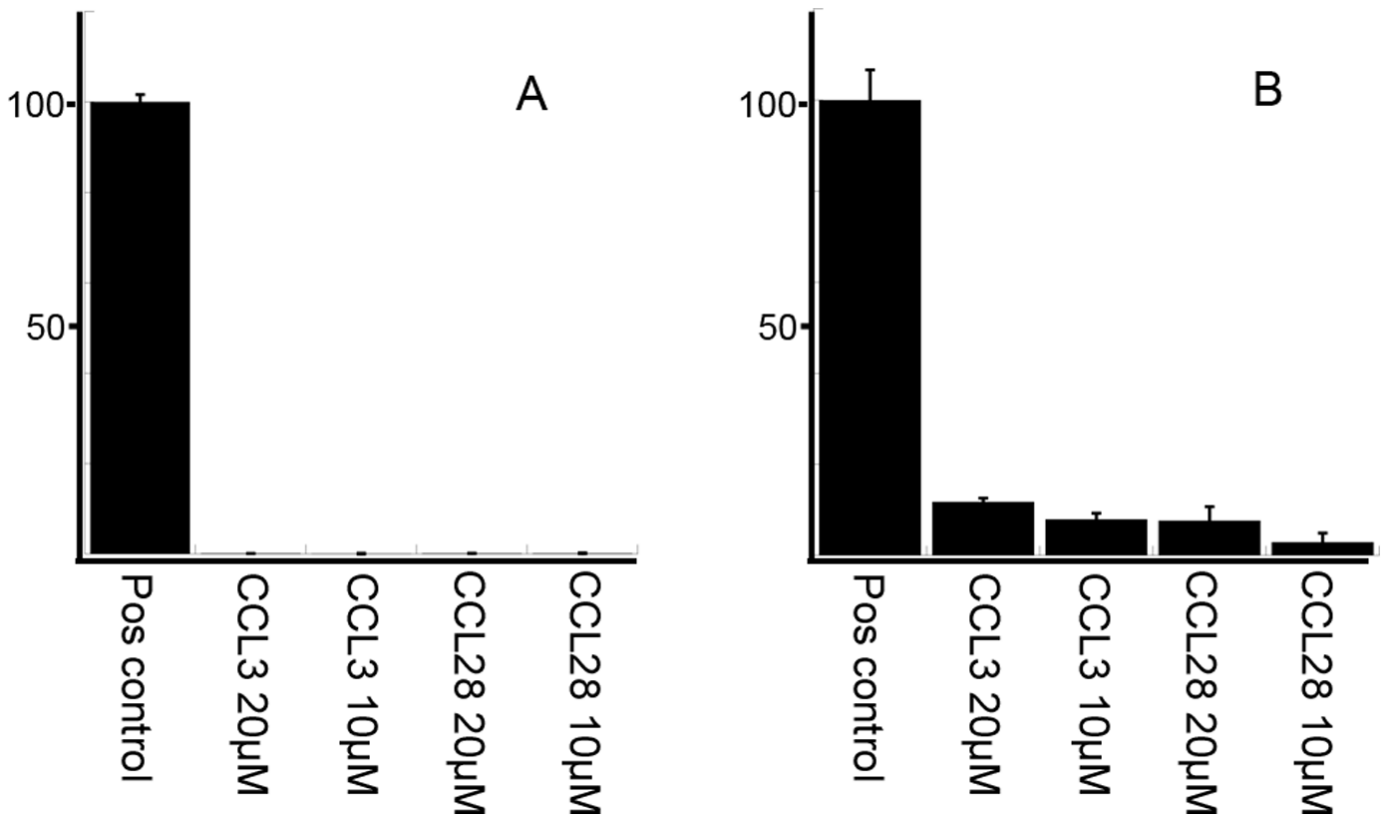
Cytotoxicity of CCL3 and CCL28 on human cells. Absorbance of haemoglobin release from erythrocytes following exposure to CCL3 and CCL28 is expressed as percent of positive control, 2% Triton X-100 (5A). Cytotoxicity on HaCaT keratinocytes were measured by cell-permeabilizing effects of CCL3 and CCL28 in the LDH-based TOX-7 kit, and absorbance expressed as percent of positive control, 1% Triton X-100 (5B). No haemolysis and very little cell-permeabilizing effects of CCL28 on keratinocytes could be seen at the concentrations used for the chemokine screen on promastigotes (10 µM). Error bars represent the standard errors of the means (n = 3).

One potential concern with membrane active peptides is cytotoxicity against human cells. We therefore tested one chemokine (CCL28) with parasiticidal activity against *L. mexicana*, and one without such activity (CCL3) for cytotoxicity against human cells. CCL28 and CCL3 at 10–20 µM were tested in a whole blood haemolysis assay and a lactate dehydrogenase (LDH) release assay on cultured keratinocytes as previously described for other antimicrobial peptides [21]. This revealed that no haemolysis was caused by CCL3, or CCL28 at concentrations of 10–20 µM, and very little LDH-release from the keratinocytes could be detected (Fig. 5). When HaCaT keratinocytes were incubated with 10 µM, a concentration shown above to be parasiticidal to the *L.mexicana* promastigotes, the LDH-release from HaCaT keratinocytes was less than 5% of the positive control.

## Discussion

Leishmaniasis is a major health problem in tropical and subtropical regions throughout the world. The clinical presentation ranges from self-healing cutaneous ulcers to systemic disease affecting internal organs, leading to death if left untreated. Treatment with the limited number of drugs available against leishmaniasis is lengthy, costly, and frequently has side effects with considerable morbidity and risks, and healing (with or without treatment) requires an appropriate immune response evoked by the host, in which chemokines are likely to play an important part. Our results indicate a direct parasiticidal effect of several chemokines on promastigotes of *Leishmania mexicana.*

AMPs are known to act in different ways on *Leishmania* parasites, either directly on plasma membranes, causing lysis, or through receptor-mediated disturbance of intracellular signalling events, sometimes leading to apoptosis [16]. There is a possibility that chemokines also use such ways to exert their action on promastigotes of *Leishmania mexicana* (Table 2). CCL28 caused substantial plasma membrane damage, which could explain why it was the chemokine which, in our screen, had the highest cytotoxic activity. As aggregation of cells was observed for most of the samples which also showed plasma membrane damage, and the clumps in SEM and light microscopy seemed to contain cell debris, a likely explanation is that lysis of the cells released cytoplasmic contents (e.g. DNA) which caused aggregation of the cells that were still intact.

CXCL9, which was also very effective in the MTT assay did not cause aggregation of cells. In preliminary experiments, in which the cells were manually counted on days 0–3 after incubation of CXCL9, the cells initially seemed intact, but were not viable, and decreased rapidly in number from day 0–3, in comparison to negative control (Table S1). CXCL9 may either cause smaller breaches in the plasma membrane, large enough to eventually kill the cell, but smaller than to allow immediate lysis, or it may exert its action through another mechanism, e.g. through induction of apoptosis. The least active of the chemokines were CCL3, CCL2, CXCL8 and CCL27. As the cationic properties of AMPs are considered important for their antimicrobial activity, the lesser antiparasitic effect of these four chemokines may partly, but not completely, be explained by the lower pI of the peptides (Table 1).

The activity of the chemokines was affected by the presence of higher salt concentrations (HBSS), or in sorbitol buffer with serum (Fig. S2, S3), but some of the chemokines seem to be effective in the combination of HBSS and serum. None of these in vitro conditions can completely accurately mimic the in vivo situation, although HBSS with serum may be considered to be the best approximation of the conditions explored. Also, the concentrations needed for chemotactic activity, as well as the concentrations found in different body fluids is usually in the nanomolar range, much lower than the ones used in our study, and in previous studies of the antibacterial activity of the chemokines [14], [15]. However, it should be noted that activity of the chemokines on the parasites will occur in the specialised microenvironment of the parasitophorous vacuole, or at the site of release of the chemokines, and these micro-physiological conditions are not known.

Although the mechanisms for the action of each chemokine needs further investigation, our results demonstrate a direct antimicrobial effect by several chemokines on *Leishmania mexicana*, something that has not been shown for parasites before. Previous studies have shown increased phagoctyosis and NO-mediated killing of protozoa in the presence of chemokines (CCL2, CCL3 and CCL5) in macrophages [2], [3], [4], [5]. Although our study did not show cytotoxic activity of CCL2 and CCL3, and has not investigated the effects on promastigotes of CCL5, it is possible that increased phagocytosis of parasites may not only be explained by chemokine-induced activation of the macrophage, but also, at least in part, by direct interaction between the chemokines and parasites prior to phagocytosis.

As an effective cellular immune response is needed to heal the *Leishmania* infection, immunomodulatory drugs are being evaluated, some of which have improved clinical outcome when combined with antimonial therapy [22]. Chemokines and chemokine receptors are also being extensively investigated as drug targets against autoimmune diseases [23]. Plasmids encoding CCL27 and CCL28 have, as adjuvants in a vaccine, increased antigen specific humoral and cellular responses to Influenza A in mice [24]. An effective prophylactic vaccine against leishmaniasis has not yet been developed, and new, better drugs for leishmaniasis are urgently needed. None of the drugs available will, on its own lead to healing of *Leishmania* infection in an immune-compromised host, as healing requires a cellular immune response to the parasites. An adequate immune response is facilitated if some of the parasite cells are dead, which could mean that even if not all parasites are killed, and perhaps fewer by physiological concentrations of chemokines, there may still be a significant activity on the parasites important for the cure of leishmaniasis. In that regard, our results indicate that topically or systemically administered chemokines, or chemokine-stimulators could be a promising target for future studies.

## Materials and Methods

### Culture Procedures

Promastigotes of the strain *L. mexicana* MHOM/GT/2001/U1103 [25], were cultured at 27°C in medium M199 (with Hank’s salts and HEPES; Invitrogen, Lidingö, Sweden) supplemented with 10% foetal calf serum (FCS), 1× BME vitamins and 25 µg/mL of Gentamicin sulphate (all from Sigma-Aldrich, Schnellendorf, Germany).

1 mL from 10 mL cultures were passaged every 7 days to new 25 mL sterile culture-flasks. Preparations of cells for the different assays followed the protocol described by Luque-Ortega et al. [19], with a few modifications. Cell counting was performed before every experiment, using a Neubauer counting chamber, and counts were always performed at least twice on the same cells.

### Assay for Antimicrobial Activity

All peptides used were recombinant human chemokines from PeproTech, London, UK. Amphotericin B (Sigma-Aldrich) was used as a positive control. Preparations of both the sorbitol buffer (280 mM D-sorbitol, 4.0 mM Na2HPO4, 1.0 mM KCl, 4.8 mM NaHCO3, 10 mM D-glucose, adjusted to pH 7.2) and HBSS (Hanks Balanced Salt Solution, containing 137 mM NaCl, 5.3 mM KCl, 0.4 mM KH2PO4, 4.2 mMNaHCO3, 0.4 mMNa2HPO4, pH 7.2, 10 mM D-glucose, adjusted to pH 7.2), followed protocols described by Luques-Ortega et. al. [19] All assays were performed on mid-exponential phase promastigote cultures harvested on day 5, centrifuged in 1,500×*g*, washed in sorbitol buffer twice, and resuspended in sorbitol buffer at a final concentration of 4×10^7^ cells/mL. Such cells were incubated at 27°C for 4 hrs in 100 µL reactions containing 10 µM of the chemokine or 5 µM of Amphotericin B in sorbitol buffer, alongside control suspensions, before use in downstream analyses. Antimicrobial activity was measured as decrease in mitochondrial metabolism in an MTT assay, essentially as described by Luque-Ortega et al. [19]. Cell death/reduction of mitochondrial activity (percentage of negative control) was calculated as follows: 100×(OD_sample_−OD_medium_ _only_/OD_negative control_−OD_medium_ _only_). The number of viable promastigotes was confirmed to correlate to the OD in this assay (Fig. S1). Statistical analysis was made using the Wilcoxon Rank-Sum test. For assays with human serum in sorbitol buffer and in HBSS, 10% fresh frozen serum from a healthy volunteer, not heat inactivated, was used (Fig. S2, S3).

### Aggregation of Cells

Cells, cultured and washed as described above, were incubated with 10 µM of the chemokines in 120 µL sorbitol buffer in 1.5 mL tubes. After careful pipetting to mix the sample, aggregation of cells was observed through the tubes, held towards a lamp. The incubated cells were also observed in light microscopy, directly after the incubation. The degree to which promastigote cells were aggregated were scored as follows; Not present (-) no change in turbidity, or present in different degrees (+) aggregation of a minority of parasites, (++) aggregation of significant proportion of parasites in light microscopy, and visible aggregation to the eye, but not complete clearing of the turbidity in the tube, (+++) aggregation of majority of parasites in light microscopy, and rapid aggregation to the eye, clearing the liquid from remaining turbidity. (Table 2).

### Flow Cytometry/entrance of Vital Dyes

Cells were incubated with chemokines in 100 µL sorbitol buffer as described above and diluted 1∶2 in PBS containing 4% propidium iodide, giving a final concentration of 2% (Calciobiochem/Merck, Nottingham, UK), incubated in the dark on ice for 30 min, and analysed with BD FACSCalibur, FL-2 (488 nm).

### Scanning Electron Microscopy

For scanning electron microscopy (SEM), one million cells, treated and incubated as described above, were centrifuged at 1,500×*g*, resuspended in fixation solution, and added to poly-L-lysine coated glass cover slips for 1 hour. The cover slip was carefully washed three times in phosphate-buffered saline (PBS), followed by incubation in 3 ml of fixation solution (4% formaldehyde and 2.5% glutaraldehyde in PBS) at room temperature for 12 hours. Fixed specimens were dehydrated for 10 min at each step of an ascending ethanol series and dried to the critical point in a Balzers critical point dryer in liquid carbon dioxide using absolute ethanol as an intermediate solvent. Samples were examined in a Jeol J-330 scanning electron microscope at an acceleration voltage of 5 kV and a working distance of 10 mm. Cells from the same incubation were cultured and counted on days 0–3.

### Cytotoxicity Assays

Haemolytic properties of the chemokines were investigated in citrate-blood, diluted 1∶1 in phosphate-buffered saline (PBS). In a v-bottom shaped 96-well plate, with 100 µL of diluted blood in each well, two of the chemokines (10 and 20 µM of CCL28 and CCL3), and Triton X-100 (2% Sigma-Aldrich, St Louis) as positive control, were incubated with careful rotation in 37°C for 1 h. The plates were then centrifuged at 800×*g* for 10 min. Haemoglobin release was measured from the supernatant (removed to another plate) at 540 nm, and expressed as the percentage of Triton X-induced haemolysis. [21].

Lactate dehydrogenase (LDH) assay was carried out on HaCaT-cells to investigate if the chemokines, in the concentrations used for the screen on the promastigotes, would have membrane-damaging properties on human keratinocytes. HaCaT-cells were grown in 96-well plates (3000 cells/well) in serum-free keratinocyte medium (SFM) supplemented with bovine pituitary extract and recombinant epidermal growth factor (BPE-rEGF) (Invitrogen) to confluence. The medium was removed, and 100 µL of 10/20 µM of CCL3/CCL28 in PBS was added in triplicate wells of the plate. The LDH-based TOX-7 kit (Sigma Aldrich, St Louis, MO) was used for quantification of LDH release from the cells, as a measurement of membrane damage to the cells. Triton X-100 (1%) was used as positive control, and LDH release expressed as percent of positive control. [21].

## Supporting Information

Figure S1 **Optical density in MTT assay corresponds to the number of viable promastigotes.** Promastigotes in a dilution series were used in the MTT assay, to confirm that absorbance correlates with the number of viable cells under the assay conditions.
(TIFF)

Figure S2 **Activity of chemokines against** ***Leishmania*** **promastigotes in the presence of serum.** MTT-assays performed for all chemokines tested in sorbitol buffer (empty columns), compared with sorbitol buffer containing 10% of human serum (filled columns), showing decreased activity of several of the chemokines in the presence of serum. Data shown are mean values of duplicate experiments, in which the activity in sorbitol buffer alone correspond to previous results.
(TIFF)

Figure S3 **Activity of chemokines against** ***Leishmania*** **promastigotes in salt-containing buffer, with or without serum.** MTT-assays performed for all screened chemokines in HBSS (empty columns), compared with HBSS containing 10% of human serum (filled columns), showing little activity of any of the chemokines in the presence of higher salt-concentrations. CXCL6, CXCL10, CCL20 and CCL28 seem to be more active in the presence of both salt and serum, than in HBSS alone. Data shown are mean values of duplicate assays.
(TIFF)

Table S1 In pilot studies, cells were prepared as described for the MTT-assay, incubated with chemokines in 27°C for 2 h, after which 50 µL of the incubation was transferred to 10 mL of culture medium (previously described), and counted manually in a Neubauer chamber day 0–3. Reactions were carried out in duplicate, and are expressed as actual numbers. The pilot studies pointed towards a differential cell survival, corresponding to reduction of mitochondrial activity later observed in MTT-assays (Fig. 1). Concentrations used were Amphotericin B (5 µM), chemokines (10 µM), and Saponin (1%).
(TIF)
