# Supplementary figures and images for: Human Chemokines as Antimicrobial Peptides with Direct Parasiticidal Effect on Leishmania mexicana In Vitro
Source: PLoS One. 2013 Mar 22;8(3):e58129. doi: 10.1371/journal.pone.0058129 (PMC3606167; doi:10.1371/journal.pone.0058129)

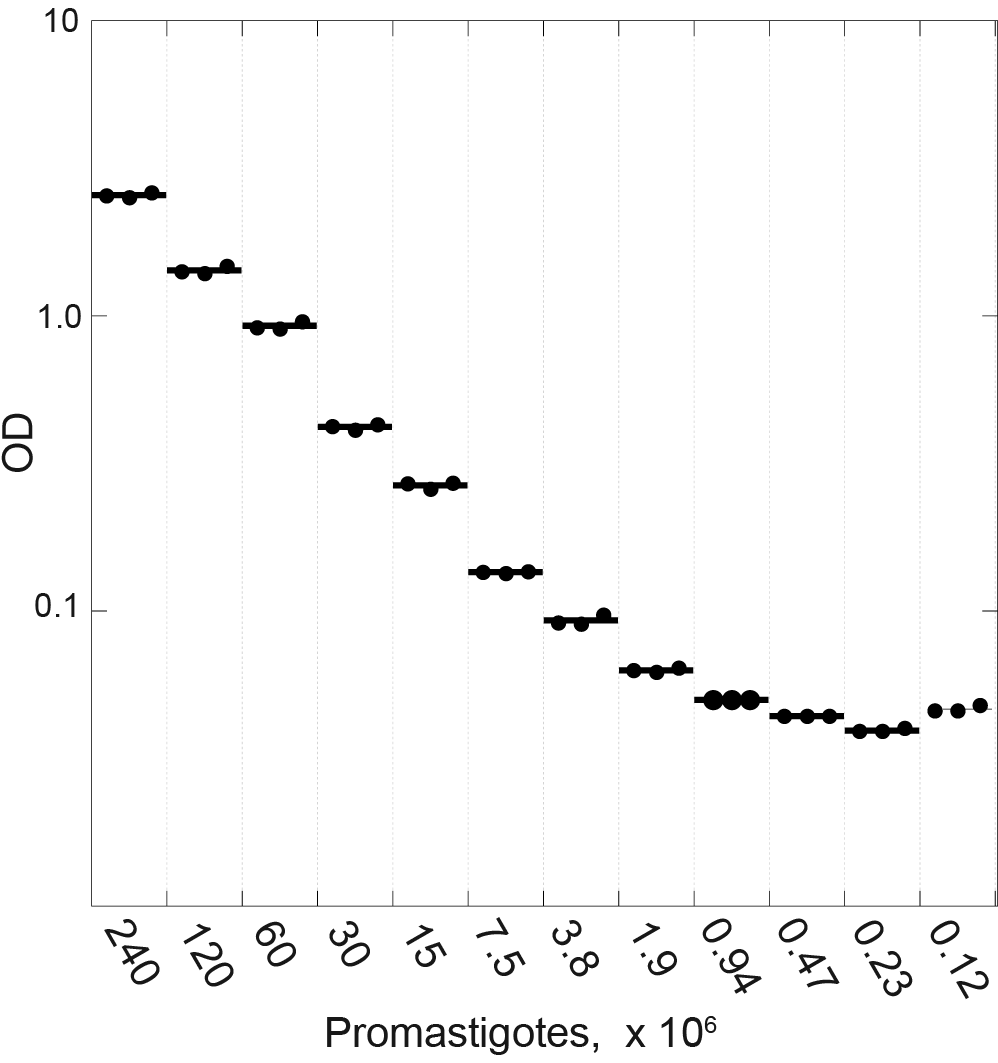

Supplement: Figure S1 — Optical density in MTT assay corresponds to the number of viable promastigotes. Promastigotes in a dilution series were used in the MTT assay, to confirm that absorbance correlates with the number of viable cells under the assay conditions. (TIFF) [file pone.0058129.s001.tif]

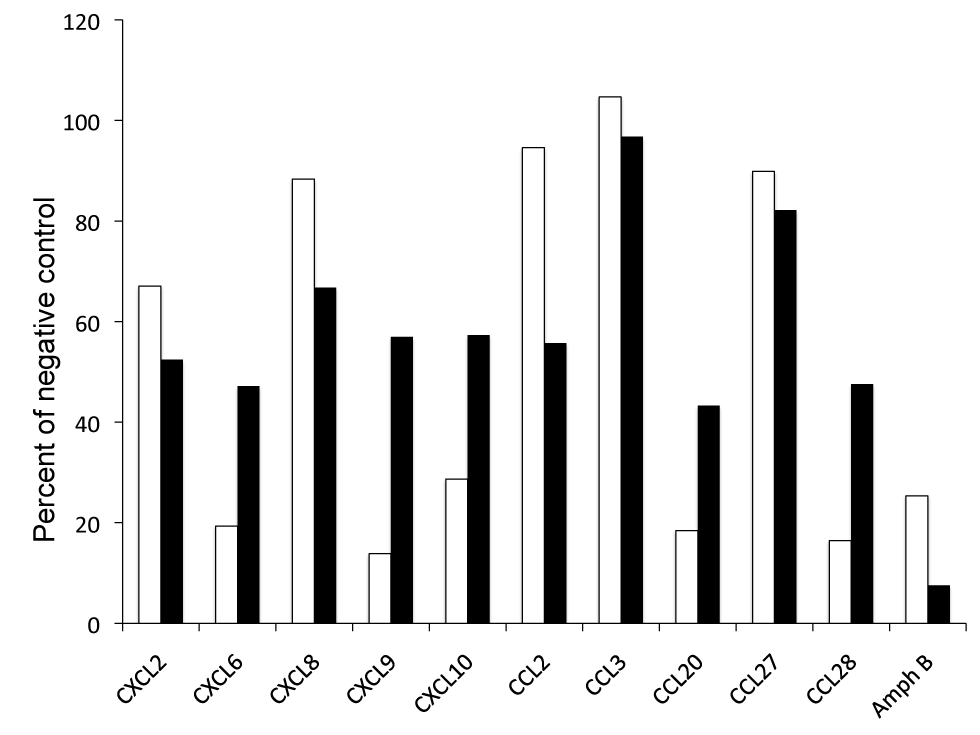

Supplement: Figure S2 — Activity of chemokines against Leishmania promastigotes in the presence of serum. MTT-assays performed for all chemokines tested in sorbitol buffer (empty columns), compared with sorbitol buffer containing 10% of human serum (filled columns), showing decreased activity of several of the chemokines in the presence of serum. Data shown are mean values of duplicate experiments, in which the activity in sorbitol buffer alone correspond to previous results. (TIFF) [file pone.0058129.s002.tif]

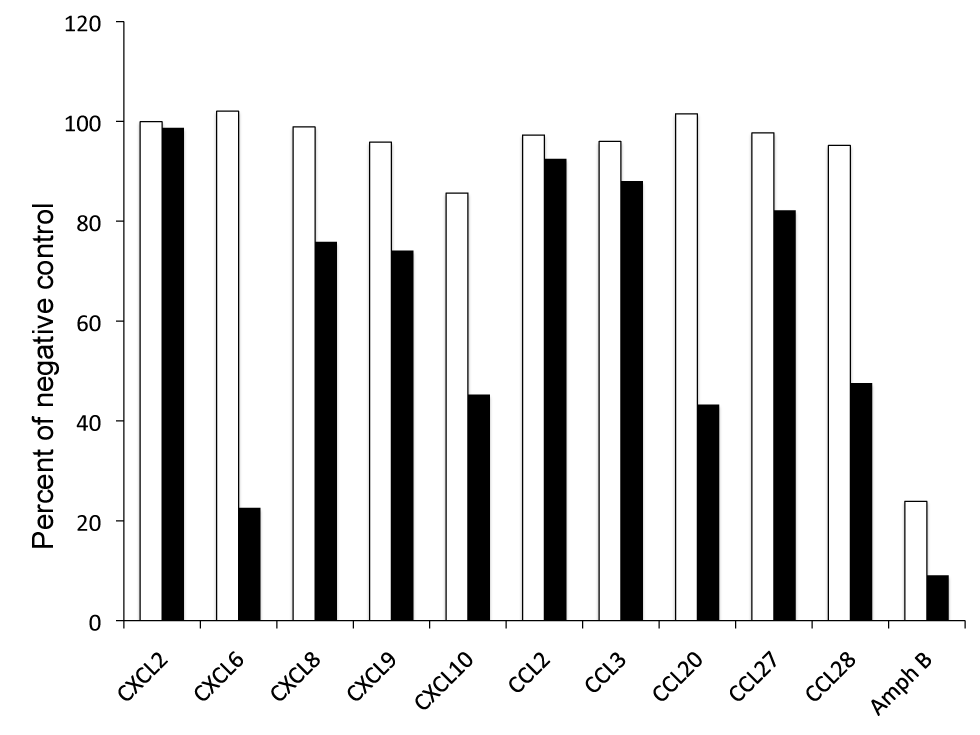

Supplement: Figure S3 — Activity of chemokines against Leishmania promastigotes in salt-containing buffer, with or without serum. MTT-assays performed for all screened chemokines in HBSS (empty columns), compared with HBSS containing 10% of human serum (filled columns), showing little activity of any of the chemokines in the presence of higher salt-concentrations. CXCL6, CXCL10, CCL20 and CCL28 seem to be more active in the presence of both salt and serum, than in HBSS alone. Data shown are mean values of duplicate assays. (TIFF) [file pone.0058129.s003.tif]

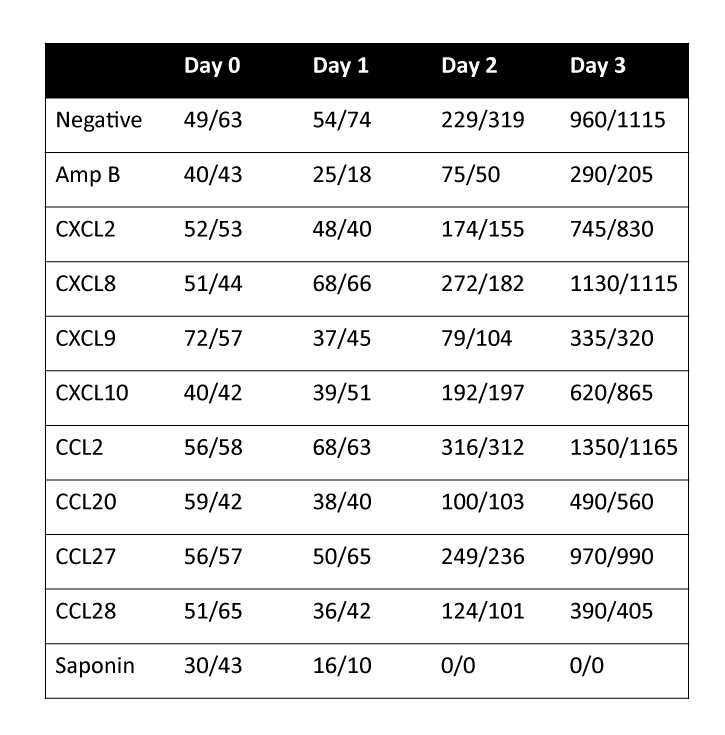

Supplement: Table S1 — In pilot studies, cells were prepared as described for the MTT-assay, incubated with chemokines in 27°C for 2 h, after which 50 µL of the incubation was transferred to 10 mL of culture medium (previously described), and counted manually in a Neubauer chamber day 0–3. Reactions were carried out in duplicate, and are expressed as actual numbers. The pilot studies pointed towards a differential cell survival, corresponding to reduction of mitochondrial activity later observed in MTT-assays (Fig. 1). Concentrations used were Amphotericin B (5 µM), chemokines (10 µM), and Saponin (1%). (TIF) [file pone.0058129.s004.tif]
